# Complete Mitochondrial Genomes and Phylogenetic Relationships of *Myotis siligorensis* and *Myotis laniger*

**DOI:** 10.1002/ece3.72094

**Published:** 2025-09-01

**Authors:** Xiao‐Die Chen, Cheng‐He Sun, Tai‐Yu Chen, Zhen‐Yu Sun, Chang‐Hu Lu

**Affiliations:** ^1^ College of Life Sciences Nanjing Forestry University Nanjing China

**Keywords:** bat, evolutionary relationships, mitochondrial genome, *Myotis*, phylogenetic analysis

## Abstract

Bats belong to the order Chiroptera, which represents the second most diverse order among mammals. Bats provide critical ecosystem services through mosquito population control, suppression of agricultural arthropod pests, pollination facilitation, and seed dispersal, while also contributing to human health preservation and economic well‐being. Moreover, they have an essential function in the ecosystem of the Earth. However, the NCBI database contains 36 mitochondrial genomes of the genus *Myotis* Kaup, 1829, and additional data are necessary to conserve the species diversity. To elucidate the phylogenetic positions of *Myotis siligorensis* (Horsfield, 1855) and *Myotis laniger* Peters, 1870 within *Myotis*, high‐throughput sequencing technology was employed in this study to obtain the mitochondrial genomes of these two species and to reconstruct a phylogenetic tree of this genus based on 13 protein‐coding genes (PCGs). The mitochondrial genomes of *M. siligorensis* and *M. laniger* were determined to be 17,067 and 17,104 base pairs in length, respectively, including 22 transfer RNA genes, 13 PCGs, 2 ribosomal RNAs, and a D‐loop. Base composition revealed a marked preference for A and T nucleotides, with the highest A + T content in the D‐loop (65.6%). Using Bayesian inference and maximum likelihood methods to reconstruct the phylogenetic tree, the results indicated that *Myotis* is monophyletic, with early speciation events occurring within the group. *M. siligorensis* , *Myotis davidii* (Peters, 1869) and *M. laniger* formed one clade, with *M. siligorensis* and *M. davidii* exhibiting a closer phylogenetic relationship with each other than with *M. laniger* . Our analytical data enhance the foundational database of *Myotis* mitochondrial genomes, enhancing our understanding of the evolutionary relationships among species within this genus. Highlighting the evolutionary relationships among different species of *Myotis* provides a solid foundation for subsequent studies on the adaptive evolution and selective pressure in bats.

## Graphical abstract

The complete mitochondrial genome of *Myotis siligorensis* and *Myotis laniger* with comments on its characteristics and phylogenetic.

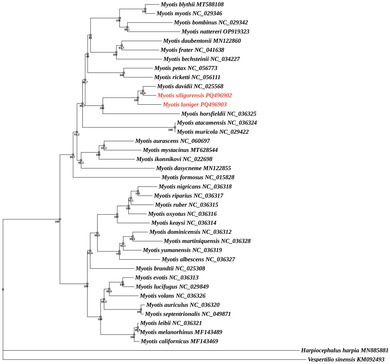

## 1 Introduction

The order Chiroptera, commonly known as bats, is classified under the class Mammalia and is divided into two suborders, Yinpterochiroptera and Yangochiroptera (Hao et al. 2024), encompassing more than 1400 species (Abdurahman et al. 2024). This makes them the second most diverse group of mammals, being the only true flying mammals. Bat species are widely distributed globally, except in Antarctica, with species diversity and numbers decreasing from low to high latitudes and increasing in tropical regions (Fang et al. 2020). Moreover, they have unique biological traits such as echolocation, flight, and longevity (Wang et al. 2017), and are known to harbor various zoonotic viruses (Yan et al. 2021). The interplay between genetic and phenotypic characteristics constitutes a significant model system (Wilkinson et al. 2021) that provides indispensable components for ecosystem functionality, and is a robust indicator of environmental health (Gibb et al. 2020).

The genus *Myotis*, belonging to the order Chiroptera, suborder Yangochiroptera, and family Vespertilionidae, according to the “bat species of the world: a taxonomic and geographic database” (Simmons and Cirranello 2025), there are about 139 species of bats. Which are found on all continents except Antarctica. These species occupy diverse ecological niches and exhibit a range of dietary specializations (Korstian et al. 2022), primarily being insectivorous, with some species exhibiting unique dietary habits, such as the fish‐eating *Myotis ricketti* (Hao 2019). Furthermore, they play crucial roles in controlling nocturnal pests and maintaining ecosystem stability (Martínez‐Fonseca et al. 2024). However, their low reproductive rates and sensitivity to environmental changes indicate that species diversity can be severely threatened by multiple factors (Bogoni et al. 2021). The challenge of classifying this genus is exacerbated by the limited availability of genetic sequence databases (Frick et al. 2020), making molecular species identification and phylogenetic analysis crucial for assessing biodiversity and formulating conservation strategies.

Advancements in modern scientific technology have led to the widespread use of molecular methods for species identification and phylogenetic analysis. Genes such as *COX1*, *CYTB*, and 16S rRNA in the mitochondrial genome have been extensively studied because of their sequence length, evolutionary rate, and inheritance patterns (Zhang et al. 2022). The mammalian mitochondrial genome has a specific structure and composition, and contains various genes and control regions, including protein‐coding genes (PCGs), ribosomal RNA (rRNA), transfer RNA (tRNA), and D‐loop (Zhang et al. 2020). Guan et al. (2025) systematically elucidated the phylogenetic relationships within the order Chiroptera by integrating 219 mitochondrial genomes (covering 187 bat species, including 54 newly sequenced) and 200 orthologous nuclear genes, and Zhang (2020) re‐sequenced the OXPHOS‐associated genes in bats, suggesting that energy metabolism genes are significant factors in the origin of bat flight. Mitochondrial genomes offer high‐resolution information, which is more conducive to constructing phylogenetic trees and analyzing species relationships (Monzel et al. 2023). However, the NCBI database currently contains 36 mitochondrial genomes of *Myotis* (Martínez‐Cárdenas et al. 2024). Accordingly, further enrichment and improvement of the mitochondrial genome database are necessary to conserve the diversity of Chiroptera species.

In this study, high‐throughput sequencing technology was employed to assess the mitochondrial genomes of two *Myotis* species and analyze their genetic compositions and structural characteristics. The obtained data were integrated with 36 published *Myotis* mitochondrial genomes in the NCBI database to ascertain their phylogenetic relationships. We constructed a phylogenetic tree to explore the phylogenetic relationships and evolutionary status of *Myotis*, providing foundational data for updating its taxonomic system. This study also provides a theoretical foundation for the effective conservation of Vespertilionidae biodiversity and the scientific evaluation of germplasm resources, advancing research and practical applications in related fields.

## 2 Materials and Methods

### 2.1 Sample Collection

All specimens in this study were collected in accordance with Chinese laws. The specimens were collected, sampled, reviewed, and approved by the Animal Ethics Committee of Nanjing Forestry University. All the experiments were conducted with respect to animal welfare and care. Specimens of *Myotis siligorensis* and *Myotis laniger* , both from the Fangshan Scenic Area in Nanjing, Jiangsu Province (118.873358° E, 31.895283° N), were sourced from roadkill carcasses obtained during the faunal surveys. Detailed information on the specimens is provided (Table 1). These specimens were preliminarily identified based on morphological analysis, which entailed an assessment of physical characteristics such as body size, facial features, ear morphology, and rostral structure, as well as measured fundamental skeletal and wing length data. *M. siligorensis* is characterized by its smaller stature, deep brown basal fur on the back, black basal fur on the ventral side, ears that fold forward to reach the rostrum, a furry snout, and smaller hind limbs (Ding et al. 2024). In contrast, *M. laniger* has a smaller physique, with dark brown fur on the head and back, blackish‐brown ventral fur, elongated ears that can reach the rostrum when folded forward, a lack of dense long fur on the snout, and relatively long hind limbs (Yang et al. 2022).

**TABLE 1:** Information on the collection of the two *Myotis* species specimens.

Specimen number	Species	Sampling time	Longitude and latitude
FS‐1	*Myotis siligorensis*	2024.09.28	118.873358° E, 31.895283° N
FS‐2	*Myotis laniger*	2024.09.28	

### 2.2 DNA Extraction

Genomic DNA was extracted from muscle tissue using DNAiso Reagent from Takara Biomedical Technology (Beijing) Co. Ltd. following the manufacturer's instructions, and stored at −20°C. To verify the accuracy of morphological identification, the primers CYTB‐F (5′‐TAG AAT ATC AGC TTT GGG TG‐3′) and CYTB‐R (5′‐AAA TCA CCG TTG TAC TTC AAC‐3′) (Xin et al. 2009) were used to amplify the *CYTB* gene in both *Myotis* specimens. The amplicons were sequenced at Nanjing Qingke Biological Company, and the results were subjected to BLAST alignment via NCBI, which confirmed that specimen FS‐1 corresponded to *M. siligorensis* and specimen FS‐2 corresponded to *M. laniger* .

### 2.3 Mitochondrial Genome Sequencing and Assembly

Muscular tissue samples were submitted to Nanjing Personalbio Technology Co. Ltd. for next‐generation sequencing. Using the Illumina NovaSeq 6000 platform, a genomic DNA library with insert fragments of 350 bp was constructed and sequenced to a depth of 8× (15G). After sequencing, the raw read data generated using the PE150 strategy were exported in the FASTQ format. Subsequently, the reads were processed using Fastp v.0.19.7 (Chen et al. 2018) to filter out adapter, highly repetitive, N‐rich, and low‐quality reads. Thereafter, the mitochondrial genomes were assembled using Geneious 2024 utilizing the “Map to Reference” tool, and the mitochondrial genome of *Myotis davidii* (NC_025568) was used as the reference sequence (Wang et al. 2016). Following assembly, ORFfinder and BLAST from the NCBI database were used to predict and annotate 13 PCGs, whereas tRNAscan‐SE identified 22 tRNA genes and their corresponding secondary structures. To ensure accuracy, all genes were manually inspected using MITOS WebServer (Bernt et al. 2013). Sequence alignment analysis was performed using MEGA v11. The mitochondrial genomes of *M. siligorensis* and *M. laniger* were visualized using the MITOfish and MITOS platforms.

### 2.4 Sequence Analysis

To ascertain the base compositions of the genomes, MEGA v11 was utilized to calculate the base content and nucleotide skew, according to the formulas “AT‐skew = (A − T)/(A + T)” and “GC‐skew = (G − C)/(G + C)”. The evolutionary characteristics of the genomes were further analyzed using DNAsp (Librado and Rozas 2009), and the rate of evolution was assessed based on the Ka/Ks ratio. The analysis of codon usage was conducted using MEGA v11 to calculate the relative synonymous codon usage (RSCU), and PhyloSuite v1.2.3 (Zhang et al. 2020) was employed to visualize the results. The Mauve multiple genome alignment method (Darling et al. 2004) within Geneious 2024 (Kearse et al. 2012) was applied to detect rearrangements and collinearity in the mitochondrial genomes, with the degree of collinearity between the two species serving as a measure of evolutionary distance, thereby allowing for analysis of the phylogenetic relationships between the species.

### 2.5 Phylogenetic Analysis

Using Bayesian inference (BI) and maximum likelihood (ML) methods, 38 effective mitochondrial genomes from *Myotis*, along with the mitochondrial genomes of *Vespertilio sinensis* (KM092493) and *Harpiocephalus harpia* (MN885881) as outgroups, were used to reconstruct the phylogenetic tree based on the 13 PCGs, as shown in Table 2. Construct phylogenetic trees using amino acid sequences. All operations were completed using PhyloSuite v1.2.3, which involved downloading GenBank sequences from NCBI, importing and curating them in PhyloSuite v1.2.3, conducting multiple sequence alignments of PCGs with MAFFT, followed by optimization with MACSE, and trimming with Gblocks. The ModelFinder (Kalyaanamoorthy et al. 2017) BIC criterion model was further selected, and BI and ML methods were applied to each dataset for phylogenetic analysis. Model selection using BIC identified GTR + F + I + G4 and GTR + F + R4 as optimal models for BI and ML analyses, respectively. The BI tree was constructed using MrBayes 3.2.6, running for 5,000,000 generations (Huelsenbeck and Ronquist 2001), and the ML tree was constructed using IQ‐TREE, with 5000 ultrafast bootstrap replicates (Nguyen et al. 2015).

**TABLE 2:** Mitochondrial genome information for the 38 *Myotis* species and two outgroup species involved in this study.

Family	Genus	Species	Size (bp)	Accession No.
Vespertilionidae	*Myotis*	*Myotis californicus*	16,972	MF143469
		*Myotis melanorhinus*	17,018	MF143489
		*Myotis dasycneme*	17,024	MN122855
		*Myotis daubentonii*	17,116	MN122860
		*Myotis blythii*	16,752	MT588108
		*Myotis mystacinus*	16,168	MT628544
		*Myotis formosus*	17,159	NC_015828
		*Myotis ikonnikovi*	16,584	NC_022698
		*Myotis brandtii*	17,470	NC_025308
		*Myotis davidii*	17,531	NC_025568
		*Myotis bombinus*	17,128	NC_029342
		*Myotis myotis*	17,213	NC_029346
		*Myotis muricola*	17,224	NC_029422
		*Myotis lucifugus*	17,038	NC_029849
		*Myotis bechsteinii*	17,151	NC_034227
		*Myotis dominicensis*	16,999	NC_036312
		*Myotis evotis*	17,039	NC_036313
		*Myotis keaysi*	17,057	NC_036314
		*Myotis ruber*	16,984	NC_036315
		*Myotis oxyotus*	17,067	NC_036316
		*Myotis riparius*	17,074	NC_036317
		*Myotis nigricans*	17,067	NC_036318
		*Myotis yumanensis*	17,268	NC_036319
		*Myotis auriculus*	17,289	NC_036320
		*Myotis leibii*	16,997	NC_036321
		*Myotis atacamensis*	17,100	NC_036324
		*Myotis horsfieldii*	17,083	NC_036325
		*Myotis volans*	17,443	NC_036326
		*Myotis albescens*	17,126	NC_036327
		*Myotis martiniquensis*	17,170	NC_036328
		*Myotis frater*	17,089	NC_041638
		*Myotis septentrionalis*	17,150	NC_049871
		*Myotis ricketti*	17,098	NC_056111
		*Myotis petax*	17,299	NC_056773
		*Myotis aurascens*	16,771	NC_060697
		*Myotis nattereri*	17,213	OP919323
		***Myotis siligorensis***	**17,067**	**PQ496902**
		***Myotis laniger***	**17,104**	**PQ496903**
	*Vespertilio*	*Vespertilio sinensis*	17,146	KM092493
	*Harpiocephalus*	*Harpiocephalus harpia*	16,446	MN885881

*Note:* Bold text specifically refers to the species information sequenced in this study.

## 3 Results

### 3.1 Genomic Structure and Base Composition

**FIGURE 1:**
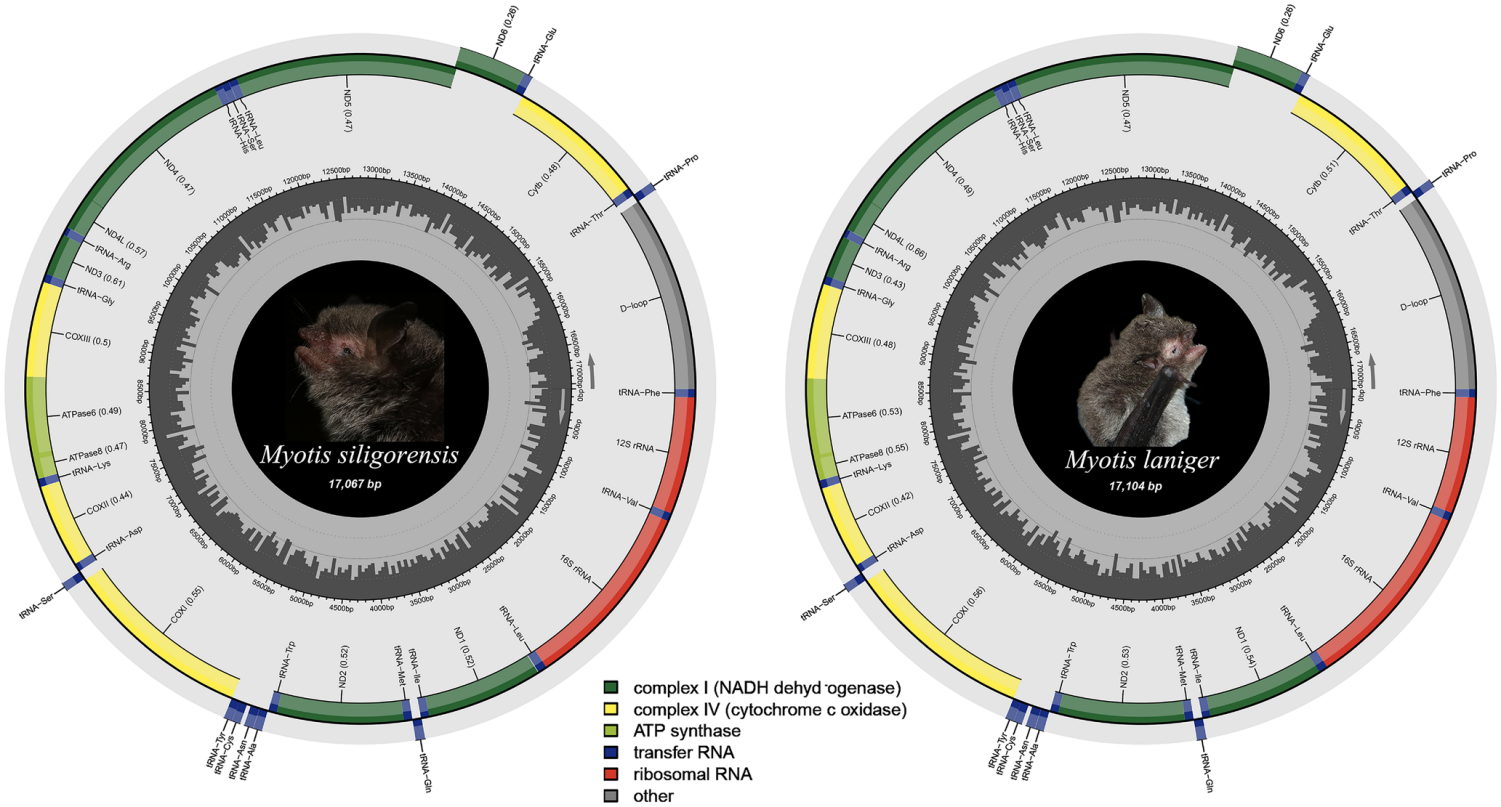
Mitochondrial genome information diagram of *Myotis siligorensis* and *Myotis laniger* . Species photographs were provided by Tai‐Yu Chen. Outer ring gene: Encoded by L‐strand. Inner circle gene: encoded by H‐strand.

The two *Myotis* mitochondrial genomes were typical double‐stranded circular molecules with sizes of 17,067 base pairs (bp) for *M. siligorensis* and 17,104 bp for *M. laniger* (Figure 1). The mitochondrial genomes of the two *Myotis* species comprised 13 PCGs, 22 tRNAs, two rRNAs, and a D‐loop. Eight tRNAs (Gln, Ala, Asn, Cys, Tyr, Ser1, Glu, and Pro) and *ND6* were encoded by the minor strand, whereas the remaining genes were located on the major strand (Table 3). The newly sequenced mitochondrial genomes of *Myotis* sp. had similar sequence lengths and gene arrangements.

**TABLE 3:** General characteristics of the mitochondrial genomes of *Myotis siligorensis* and *Myotis laniger* .

Gene	Position		Size (bp)	Intergenic nucleotides	Codon		Strand
	From	To			Initiation	Termination	
*tRNA‐Phe*	1/1	69/68	69/68	0/0			+/+
*12S rRNA*	70/69	1034/1033	965/965	0/0			+/+
*tRNA‐Val*	1035/1034	1102/1102	68/69	0/0			+/+
*16S rRNA*	1103/1103	2666/2669	1564/1567	0/0			+/+
*tRNA‐Leu*	2667/2670	2741/2744	75/75	0/0			+/+
*ND1*	2747/2750	3702/3705	956/956	5/5	ATG/ATG	TA/TA	+/+
*tRNA‐Ile*	3703/3706	3771/3774	69/69	0/0			+/+
*tRNA‐Gln*	3769/3772	3842/3845	74/74	−3/−3			−/−
*tRNA‐Met*	3843/3846	3912/3915	70/70	0/0			+/+
*ND2*	3913/3916	4954/4957	1042/1042	0/0	ATT/ATT	T/T	+/+
*tRNA‐Trp*	4955/4958	5022/5025	68/68	0/0			+/+
*tRNA‐Ala*	5028/5031	5096/5099	69/69	5/5			−/−
*tRNA‐Asn*	5098/5101	5170/5173	73/73	1/1			−/−
*tRNA‐Cys*	5203/5206	5268/5271	66/66	32/32			−/−
*tRNA‐Tyr*	5269/5272	5335/5339	67/68	0/0			−/−
*COX1*	5337/5341	6881/6885	1545/1545	1/1	ATG/ATG	TAA/TAA	+/+
*tRNA‐Ser1*	6895/6899	6963/6967	69/69	13/13			−/−
*tRNA‐Asp*	6971/6975	7037/7041	67/67	7/7			+/+
*COX2*	7038/7042	7721/7725	684/684	0/0	ATG/ATG	TAA/TAA	+/+
*tRNA‐Lys*	7725/7729	7792/7796	68/68	3/3			+/+
*ATP8*	7794/7798	7997/8004	204/207	1/1	ATG/ATG	TAA/TAG	+/+
*ATP6*	7955/7959	8634/8638	680/680	−43/−46	ATG/ATG	TA/TA	+/+
*COX3*	8635/8639	9418/9422	784/784	0/0	ATG/ATG	T/T	+/+
*tRNA‐Gly*	9419/9423	9487/9491	69/69	0/0			+/+
*ND3*	9488/9492	9834/9838	347/347	0/0	ATA/ATA	TA/TA	+/+
*tRNA‐Arg*	9835/9839	9903/9907	69/69	0/0			+/+
*ND4L*	9905/9909	10,201/10,205	297/297	1/1	ATG/ATG	TAA/TAA	+/+
*ND4*	10,195/10,199	11,572/11,576	1378/1378	−7/−7	ATG/ATG	T/T	+/+
*tRNA‐His*	11,573/11,577	11,640/11644	68/68	0/0			+/+
*tRNA‐Ser2*	11,641/11,645	11,700/11,703	60/59	0/0			+/+
*tRNA‐Leu2*	11,702/11,705	11,771/11,774	70/70	1/1			+/+
*ND5*	11,772/11,775	13,592/13,595	1821/1821	0/0	ATA/ATA	TAA/TAA	+/+
*ND6*	13,576/13,579	14,103/14,106	528/528	−17/−17	ATG/ATG	TAA/TAA	−/−
*tRNA‐Glu*	14,104/14,107	14,172/14,175	69/69	0/0			−/−
*CYTB*	14,180/14,183	15,319/15,322	1140/1140	7/7	ATG/ATG	AGA/AGA	+/+
*tRNA‐Thr*	15,320/15,323	15,389/15,393	70/71	0/0			+/+
*tRNA‐Pro*	15,389/15,393	15,454/15,458	66/66	−1/−1			−/−
D‐loop	15,455/15,459	17,067/17,104	1613/1646	0/0			

Both mitochondrial genomes had five gene overlaps and 12 intergenic spaces (Table 3). The overlaps were situated between *tRNA‐Ile* and *tRNA‐Gln* (3 bp), *ATP8* and *ATP6* (43 bp for *M. siligorensis* and 46 bp for *M. laniger* ), *ND4L* and *ND4* (7 bp), *ND5* and *ND6* (17 bp), and *tRNA‐Thr* and *tRNA‐Pro* (1 bp). The longest and shortest sequences were 46 and 1 bp, respectively. The intergenic spaces were determined to be located between *tRNA‐Leu* and *ND1* (5 bp).

### 3.2 Nucleotide Composition

**FIGURE 2:**
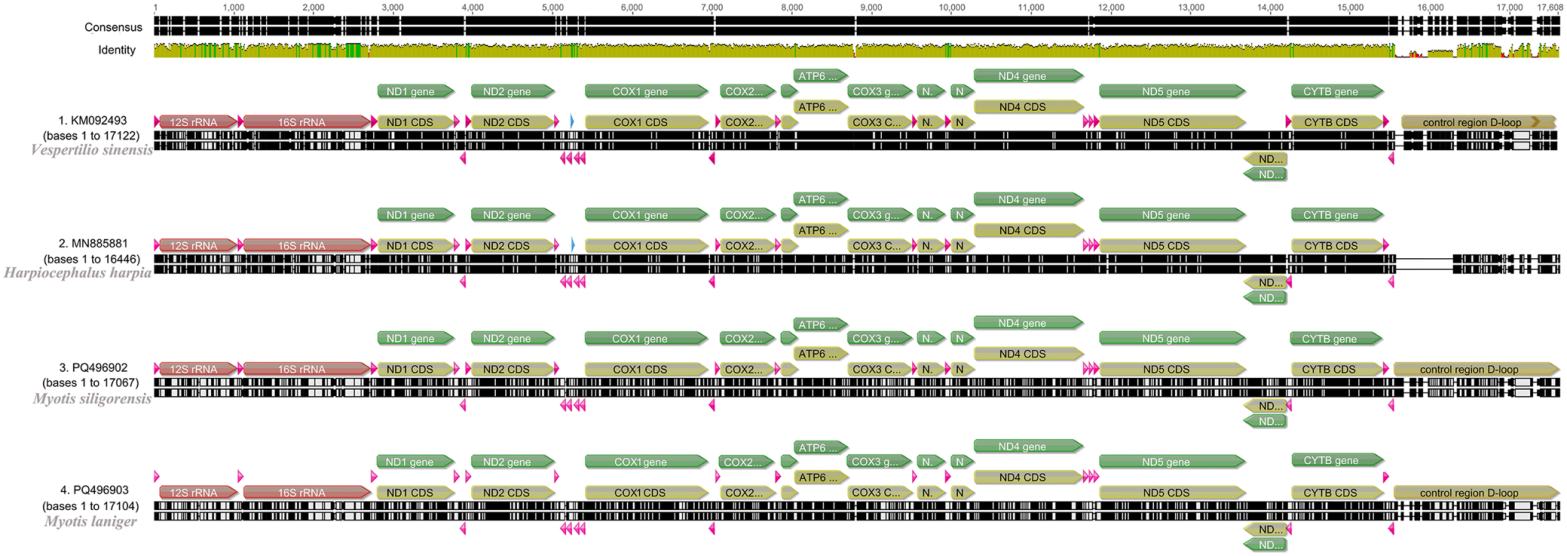
Collinearity analysis comparing *Myotis siligorensis* , *Myotis laniger* , and the outgroup species *Vespertilio sinensis* and *Harpiocephalus harpia* .

Nucleotide composition analysis of the two *Myotis* bat species revealed a greater AT skew than GC skew in the mitochondrial genomes (Table 4), indicating a preference for A and T nucleotides. Both mitochondrial genomes exhibited an AT bias (A + T > G + C), which was also evident in PCGs, rRNAs, and D‐loops. The highest A + T content in the D‐loop was observed in *M. siligorensis* (65.6%), whereas the highest A + T content within the tRNAs was observed in *M. laniger* (64.7%). The AT skew ranged from −0.019 to 0.215, and the GC skew ranged from −0.285 to 0.076 in the two mitochondrial genomes (Table 4). Gene collinearity analysis (Figure 2) demonstrated a high degree of similarity between the genomes of the two *Myotis* species, with no evidence of gene rearrangement.

**TABLE 4:** Structural components and skew of the mitochondrial genomes of the two *Myotis* species.

Species	Region	Size (bp)	A	T(U)	C	G	A + T%	AT‐Skew	GC‐Skew
*Myotis siligorensis*	Total genome	17,067	33.7	29.8	23.4	13.1	63.5	0.061	−0.282
	PCGs	11,406	31.1	32.0	23.7	13.2	63.1	−0.014	−0.285
	tRNAs	1513	32.7	31.6	16.6	19.2	64.3	0.017	0.073
	rRNAs	2529	38.0	25.4	19.8	16.8	63.4	0.199	−0.082
	D‐loop	1613	38.1	27.5	23.1	11.3	65.6	0.162	−0.343
*Myotis laniger*	Total genome	17,104	33.4	29.6	23.6	13.4	63.0	0.060	−0.276
	PCGs	11,400	30.7	32.0	23.9	13.4	62.7	−0.019	−0.282
	tRNAs	1514	33.0	31.7	16.3	19.0	64.7	0.020	0.076
	rRNAs	2532	38.3	24.9	20.1	16.7	63.2	0.212	−0.092
	D‐loop	1646	37.1	27.1	23.4	12.5	64.2	0.156	−0.304

### 3.3 Protein‐Coding Genes and Codon Usage Patterns

**FIGURE 3:**
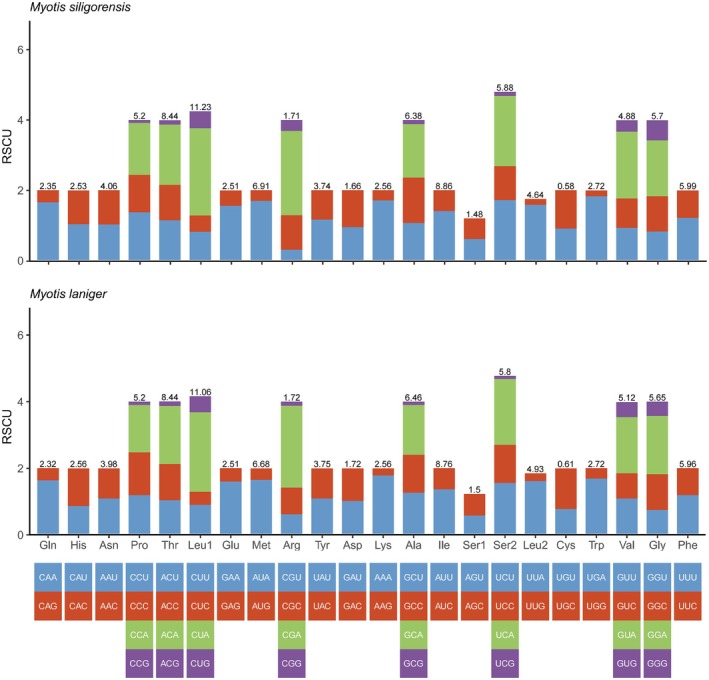
Relative synonymous codon usage analysis results for two *Myotis* species. The x‐axis refers to 64 synonymous codons (arranged in the order of amino acids, and termination codons are usually excluded).

**FIGURE 4:**
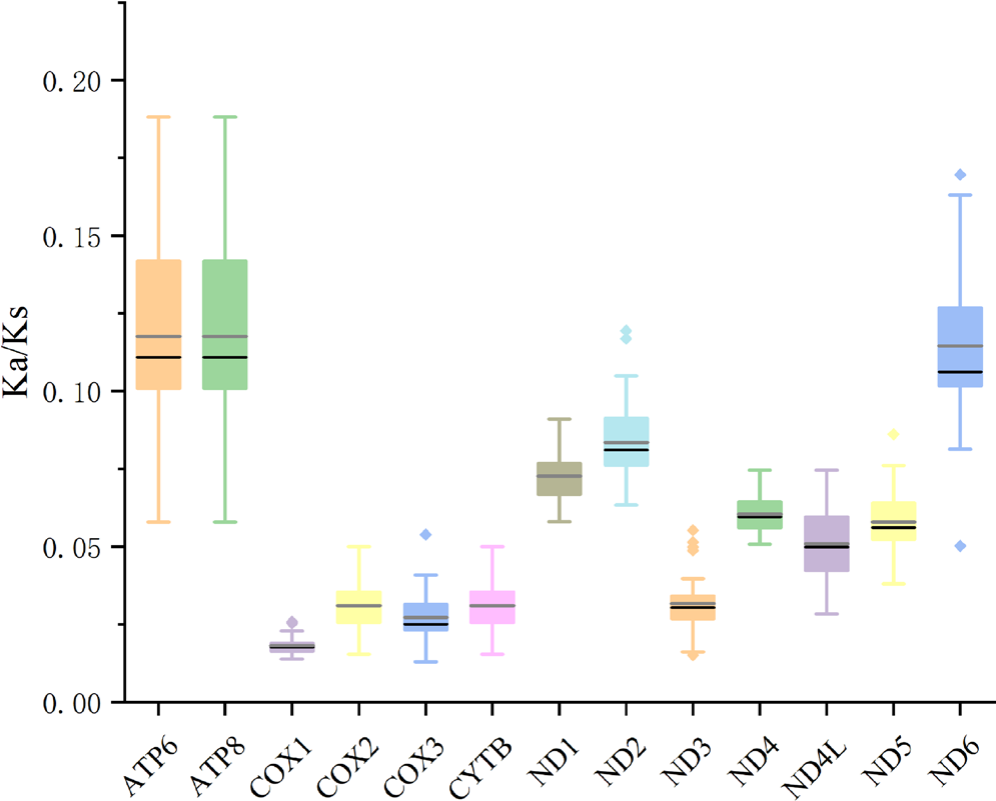
Analysis of Ka/Ks ratios for 13 protein‐coding genes across 38 species of *Myotis*. Solid gray and black lines indicate the mean and median values, respectively.

The aggregate lengths of the 13 PCGs in the mitochondrial genomes of *M. siligorensis* and *M. laniger* were 11,406 bp and 11,400 bp, constituting 63.1% and 62.7% of the mitochondrial genome sequence length, respectively. Except for *ND6*, which was encoded by the minor strand, all the other PCGs were encoded by the major strand (Table 3). Among the PCGs of the two mitochondrial genomes, *ND2* was initiated with an ATT codon, *ND3* and *ND5* were initiated with an ATA codon, and all other start codons were ATG (Table 3). Five types of termination codons were present in the two *Myotis* species, namely TA, T, TAA, AGA, and TAG, with the following distributions: *ND1*, *ATP6*, and *ND3* used TA as the termination codon; *ND2*, *COX3*, and *ND4* used T; *COX1*, *COX2*, *ND4L*, *ND5*, *ND6*, and *ATP8* of *M. siligorensis* used TAA; *CYTB* terminated with AGA; and *ATP8* of *M. laniger* terminated with TAG. The TAA termination codon was the most frequent, whereas TAG was the least frequent (Table 3). Using RSCU analysis, we investigated the codon usage patterns of two *Myotis* mitochondrial genomes (Figure 3) and found a high degree of similarity. The codon CUA was associated with the highest RSCU values in both *M. siligorensis* (2.49) and *M. laniger* (2.38), whereas the codon UCG was associated with the lowest RSCU values, at 0.11 and 0.09, respectively (Tables 5 and 6). The evolutionary patterns of the two *Myotis* species were analyzed based on the Ka/Ks ratios. All PCGs had Ka/Ks ratios less than 1 (Figure 4), with *ATP8* having the highest Ka/Ks ratio at 0.118 and *COX1* having the lowest at 0.018.

**TABLE 5:** Frequency of *Myotis siligorensis* codon usage.

Codon	Count	RSCU	Codon	Count	RSCU	Codon	Count	RSCU	Codon	Count	RSCU
UUU(F)	139	1.22	UCU(S)	80	1.72	UAU(Y)	83	1.17	UGU(C)	10	0.91
UUC(F)	88	0.78	UCC(S)	45	0.97	UAC(Y)	59	0.83	UGC(C)	12	1.09
UUA(L)	160	1.59	UCA(S)	93	2	UAA(*)	6	3.43	UGA(W)	94	1.83
UUG(L)	16	0.16	UCG(S)	5	0.11	UAG(*)	0	0	UGG(W)	9	0.17
CUU(L)	82	0.82	CCU(P)	68	1.38	CAU(H)	50	1.04	CGU(R)	5	0.31
CUC(L)	46	0.46	CCC(P)	52	1.06	CAC(H)	46	0.96	CGC(R)	16	0.98
CUA(L)	250	2.49	CCA(P)	73	1.48	CAA(Q)	74	1.66	CGA(R)	39	2.4
CUG(L)	48	0.48	CCG(P)	4	0.08	CAG(Q)	15	0.34	CGG(R)	5	0.31
AUU(I)	237	1.41	ACU(T)	92	1.15	AAU(N)	79	1.03	AGU(S)	29	0.62
AUC(I)	99	0.59	ACC(T)	81	1.01	AAC(N)	75	0.97	AGC(S)	27	0.58
AUA(M)	223	1.7	ACA(T)	137	1.71	AAA(K)	83	1.71	AGA(*)	1	0.57
AUG(M)	39	0.3	ACG(T)	10	0.12	AAG(K)	14	0.29	AGG(*)	0	0
GUU(V)	43	0.93	GCU(A)	65	1.07	GAU(D)	30	0.95	GGU(G)	45	0.83
GUC(V)	39	0.84	GCC(A)	78	1.29	GAC(D)	33	1.05	GGC(G)	54	1
GUA(V)	88	1.9	GCA(A)	92	1.52	GAA(E)	74	1.56	GGA(G)	86	1.59
GUG(V)	15	0.32	GCG(A)	7	0.12	GAG(E)	21	0.44	GGG(G)	31	0.57

**TABLE 6:** Codon usage frequencies for *Myotis laniger* .

Codon	Count	RSCU	Codon	Count	RSCU	Codon	Count	RSCU	Codon	Count	RSCU
UUU(F)	134	1.19	UCU(S)	72	1.56	UAU(Y)	78	1.1	UGU(C)	9	0.78
UUC(F)	92	0.81	UCC(S)	53	1.15	UAC(Y)	64	0.9	UGC(C)	14	1.22
UUA(L)	164	1.62	UCA(S)	91	1.97	UAA(*)	5	2.86	UGA(W)	87	1.69
UUG(L)	23	0.23	UCG(S)	4	0.09	UAG(*)	1	0.57	UGG(W)	16	0.31
CUU(L)	91	0.9	CCU(P)	59	1.2	CAU(H)	42	0.87	CGU(R)	10	0.62
CUC(L)	40	0.4	CCC(P)	63	1.28	CAC(H)	55	1.13	CGC(R)	13	0.8
CUA(L)	240	2.38	CCA(P)	70	1.42	CAA(Q)	72	1.64	CGA(R)	40	2.46
CUG(L)	48	0.48	CCG(P)	5	0.1	CAG(Q)	16	0.36	CGG(R)	2	0.12
AUU(I)	227	1.37	ACU(T)	83	1.04	AAU(N)	83	1.1	AGU(S)	27	0.58
AUC(I)	105	0.63	ACC(T)	87	1.09	AAC(N)	68	0.9	AGC(S)	30	0.65
AUA(M)	210	1.66	ACA(T)	139	1.74	AAA(K)	87	1.79	AGA(*)	1	0.57
AUG(M)	43	0.34	ACG(T)	11	0.14	AAG(K)	10	0.21	AGG(*)	0	0
GUU(V)	53	1.09	GCU(A)	78	1.27	GAU(D)	33	1.02	GGU(G)	40	0.75
GUC(V)	37	0.76	GCC(A)	70	1.14	GAC(D)	32	0.98	GGC(G)	58	1.08
GUA(V)	82	1.69	GCA(A)	91	1.49	GAA(E)	76	1.6	GGA(G)	93	1.74
GUG(V)	22	0.45	GCG(A)	6	0.1	GAG(E)	19	0.4	GGG(G)	23	0.43

### 3.4 rRNA, tRNA, and D‐Loops

The two rRNAs (*12S rRNA* and *16S rRNA*) were located between *tRNA‐Phe* and *tRNA‐Val*, and between *tRNA‐Val* and *tRNA‐Leu*, respectively (Table 3). The sizes of *12S rRNA* were 965 and 965 bp, and those of *16S rRNA* were 1564 and 1567 bp, respectively. The sizes of the 22 tRNAs ranged from 60 to 75 bp in *M. siligorensis* and from 59 to 75 bp in *M. laniger* , with 14 tRNAs originating from the major strand and eight from the minor strand (Table 3). The total length of the tRNAs was 1513 bp in *M. siligorensis* and 1514 bp in *M. laniger* (Table 4), accounting for 8.87% and 8.85% of the mitochondrial genomes, respectively. The D‐loop was situated between the *tRNA‐Pro* and *tRNA‐Phe* genes (Table 3), with sizes of 1613 and 1646 bp in the two species, respectively (Table 4).

### 3.5 Phylogenetic Relationships

**FIGURE 5:**
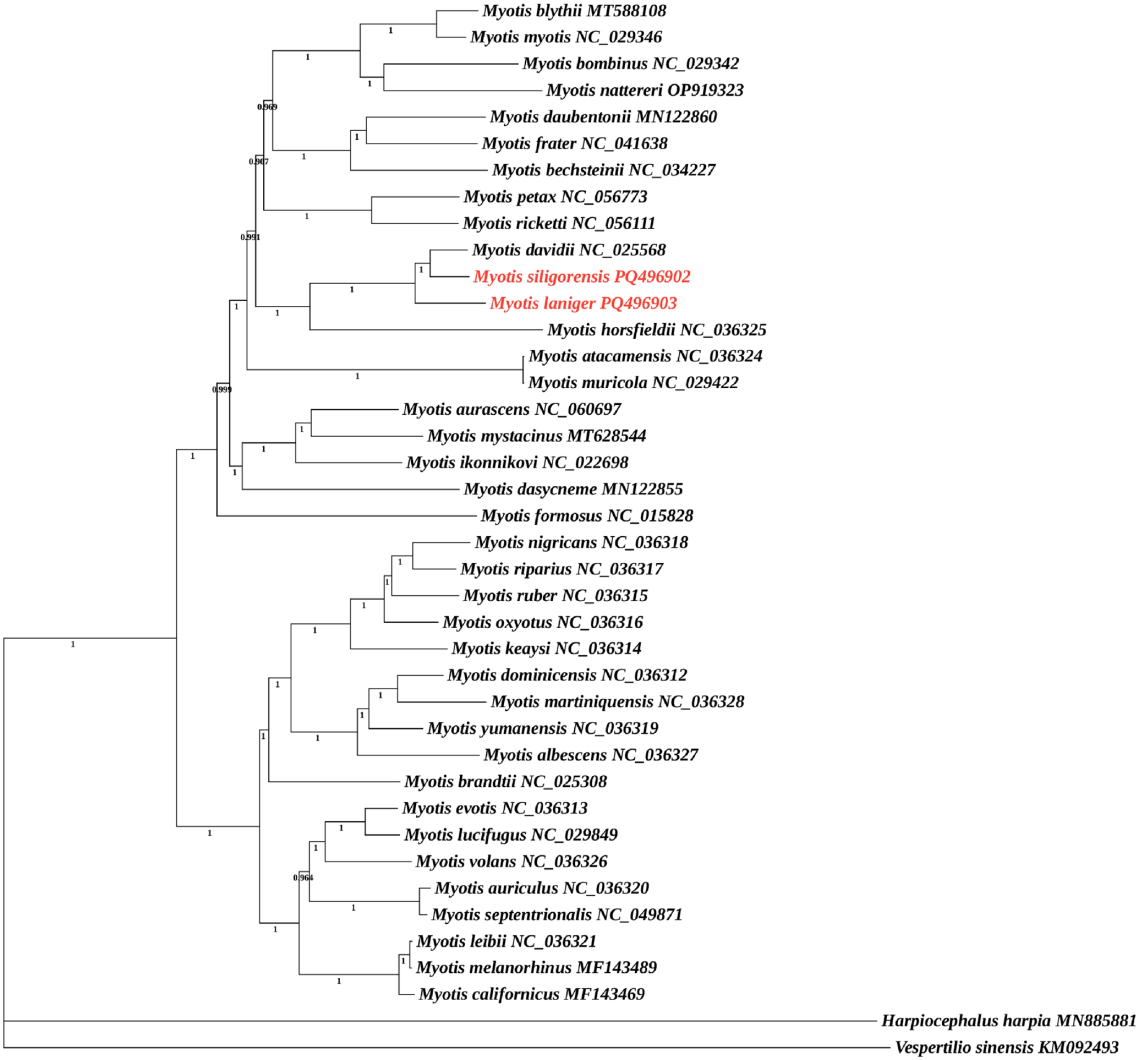
Bayesian inference (BI) tree constructed from 13 protein‐coding genes of 40 species. The values on the branches represent posterior probabilities (PP), with only nodes having PP ≥ 0.95 displayed. The numbers following the species names are GenBank accession numbers. The outgroups are *Harplocephalus harpia* (MN885881) and *Vespertilio sinensis* (KM092493).

**FIGURE 6:**
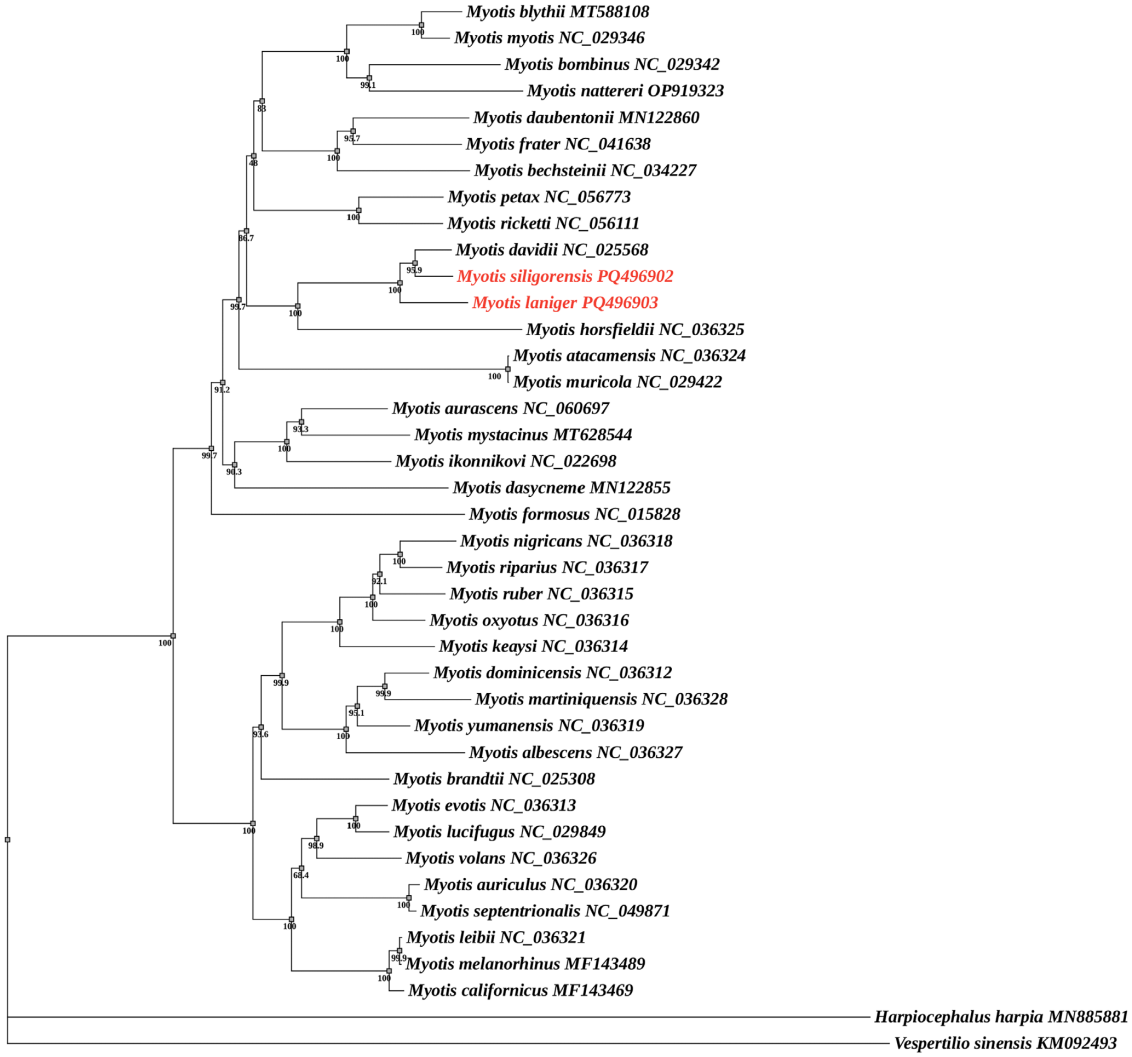
Maximum likelihood (ML) tree constructed from 13 protein‐coding genes of 40 species. The values on the branches represent bootstrap support (BS), with only nodes having BS ≥ 70% displayed. The numbers following the species names are GenBank accession numbers. The outgroups are *Harplocephalus harpia* (MN885881) and *Vespertilio sinensis* (KM092493).

Using *V. sinensis* and *H. harpia* as outgroups and integrating the genetic sequences of the two *Myotis* species sequenced in this study, phylogenetic analysis was conducted on the 36 *Myotis* species based on the 13 PCGs retrieved from GenBank (Table 2). The results indicate that the BI (Figure 5) and ML trees (Figure 6) were fundamentally consistent in their topological structures, with each branch exhibiting robust support values. The 38 *Myotis* species analyzed in this study formed a monophyletic group. Moreover, intrageneric speciation events in *Myotis* occurred early in its evolution, resulting in its divergence into two major clades. Further, *M. siligorensis* and *M. laniger* were clustered with *Myotis davidii* within the same clade, with a closer phylogenetic relationship observed between *M. siligorensis* and *M. davidii* compared to that with *M. laniger* .

## 4 Discussion

*Myotis* serves as an exemplary model for the study of species formation, with the characteristics of long‐living mammals, making it an important model for research on aging. The mitochondrial genome of *Myotis* is a vital resource for molecular phylogenetic and evolutionary (Cooper et al. 2024). This study represents the first acquisition and characterization of the mitochondrial genomes of *M. siligorensis* and *M. laniger* , enriching the mitochondrial genomic information for these species and clarifying their phylogenetic status and kinship within *Myotis*. These findings provide foundational data pertaining to the systematic molecular evolution of the Vespertilionidae family for subsequent research.

Phylogenetic analysis corroborated the evolutionary relationships within the genus *Myotis*, confirming that it is a monophyletic group, with all species sharing a recent common ancestor, which is consistent with previous findings (Vargas‐Trejo et al. 2023; Korstian et al. 2022). The genus *Myotis* underwent early divergence into two major clades, with close relationships observed between *M. davidii* / *Myotis myotis* and *Myotis blythii* / *M. ricketti* (Hao et al. 2024). It is noteworthy that resolving the phylogenetic relationships of closely related species often poses significant challenges, as different genetic markers may support conflicting topologies due to variations in their evolutionary characteristics. For instance, in the study by Liu et al. (2023) based on the mitochondrial *CYTB* gene, *M. davidii* exhibited a distant relationship with *M. siligorensis*, whereas our analysis combining 13 protein‐coding genes supported a closer phylogenetic affinity between *M. davidii* and *M. siligorensis* . Some scholars have pointed out that the Quaternary glaciations influenced clade divergence, particularly in *M. siligorensis* and *M. davidii* (Zhang and Feng 2011; Fu and Wen 2023). Additionally, phylogenetic analyses by other researchers have revealed key speciation events in the genus *Myotis*, including the split between Old World and New World lineages, as well as the later formation of the Nearctic/Neotropical subclade prior to the emergence of the Isthmus of Panama (Lack et al. 2010; Jiang et al. 2019; Luo et al. 2023).

The limited availability of genomic data for bat species, particularly those within the *Myotis* genus, has hindered in‐depth research into their evolutionary processes (Gutiérrez et al. 2024). Previous studies on bats have primarily focused on acquiring and comparing genomic data to establish correlations for multifaceted adaptive research (Thomas 2010). Although this study provides new mitochondrial genome data, contributing to a better understanding of interspecies relationships and offering insights for biodiversity conservation and germplasm resource assessment, the lack of more comprehensive genomic information remains a limitation. Future efforts should prioritize supplementing and refining genomic datasets to enhance our understanding of bat evolution, speciation, and adaptive mechanisms.

## Author Contributions

**Xiao‐Die Chen:** formal analysis (lead), writing – original draft (lead), writing – review and editing (lead). **Cheng‐He Sun:** conceptualization (equal), data curation (equal), formal analysis (equal), funding acquisition (equal), methodology (equal), writing – review and editing (equal). **Tai‐Yu Chen:** investigation (lead), writing – review and editing (supporting). **Zhen‐Yu Sun:** investigation (lead), writing – review and editing (supporting). **Chang‐Hu Lu:** funding acquisition (lead), writing – review and editing (supporting).

## Conflicts of Interest

The authors declare no conflicts of interest.

## Data Availability

The data presented in this study were deposited in the NCBI repository (accession numbers PQ496902 and PQ496903).
